# Molecular characterization and functional analysis of cytochrome P450-mediated detoxification *CYP302A1* gene involved in host plant adaptation in *Spodoptera frugieprda*

**DOI:** 10.3389/fpls.2022.1079442

**Published:** 2023-01-25

**Authors:** Muhammad Hafeez, Xiaowei Li, Limin Chen, Farman Ullah, Jun Huang, Zhijun Zhang, Jinming Zhang, Junaid Ali Siddiqui, Shu-xing Zhou, Xiao-yun Ren, Muhammad Imran, Mohammed A. Assiri, Yonggen Lou, Yaobin Lu

**Affiliations:** ^1^ State Key Laboratory of Rice Biology and Ministry of Agriculture Key Lab of Molecular Biology of Crop Pathogens and Insects, Institute of Insect Sciences, Zhejiang University, Hangzhou, China; ^2^ State Key Laboratory for Managing Biotic and Chemical Threats to the Quality and Safety of Agro-products, Institute of Plant Protection and Microbiology, Zhejiang Academy of Agricultural Sciences, Hangzhou, China; ^3^ Integrated Plant Protection Center, Lishui Academy of Agricultural and Forestry Sciences, Lishui, China; ^4^ Department of Plant Biosecurity, College of Plant Protection, China Agricultural University, Beijing, China; ^5^ College of Agriculture, College of Tobacco Science, Guizhou University, Guiyang, China; ^6^ Department of Chemistry, Faculty of Science, King Khalid University, Abha, Saudi Arabia

**Keywords:** P450 detoxification enzyme, spodoptera frugiperda, CYP302a1, host adaptation, RNAi

## Abstract

The fall armyworm (FAW) *Spodoptera frugiperda* is a destructive and polyphagous pest of many essential food crops including maize and rice. The FAW is hard to manage, control, or eradicate, due to its polyphagous nature and voracity of feeding. Here, we report the characterization and functional analysis of the detoxification gene *CYP302A1* and how *S. frugieprda* larvae use a detoxification mechanism to adapt host plants. Results demonstrated that *CYP302A1* expression levels were much higher in midgut tissue and the older *S. frugiperda* larvae. Our current studies revealed the enhanced P450 activity in the midguts of *S. frugiperda* larvae after exposure to rice plants as compared to corn plants and an artificial diet. Furthermore, higher mortality was observed in PBO treated larvae followed by the exposure of rice plants as compared to the corn plant. The dsRNA-fed larvae showed downregulation of CYP302A1 gene in the midgut. At the same time, higher mortality, reduced larval weight and shorter developmental time was observed in the dsRNA-fed larvae followed by the exposure of rice plant as compared to the corn plant and DEPC-water treated plants as a control. These results concluded that the inducible P450 enzyme system and related genes could provide herbivores with an ecological opportunity to adapt to diverse host plants by utilizing secondary compounds present in their host plants.

## Introduction

The interaction between herbivorous pests and their host plants is frequently cited as a textbook example of co-evolution (Vandenhole et al., 2021). Feeding on plants is complicated because plants have developed a wide range of morphological and chemical defensive line tactics. To avoid or diminish feeding injury, most plants induce complex chemical defense combinations to withstand insect attack (Büchel et al., 2016; Lackus et al., 2018). These chemical compounds may serve as defensive due to their unpleasant flavors and odors, or they may be toxic or mitigate the digestibility of plant tissues (Biere et al., 2004; Rehman et al., 2012). Nonetheless, identification and response to phytochemicals (allelochemicals) released by plants to defend themselves against herbivores is an important aspect of arthropod-plant interactions (Janz, 2011; Suchan and Alvarez, 2015; Vandenhole et al., 2021). The study of the interaction between plant secondary chemistry and insect herbivores is essential in the development of a successfully integrated pest management plan.

To survive, different tactics have been evolved by adapted herbivores to deal with the defense compound present in their host plants (Heidel-Fischer and Vogel, 2015a; Rashid War et al., 2018; Yang et al., 2022). Similarly, some herbivores insects metabolize and excrete plant defense compounds they consume, but others store them in their bodies to defend themselves against all-natural enemies (Petschenka and Agrawal, 2016; Heckel, 2018). Metabolic detoxification of plant toxins is the primary strategy of herbivores occurring in three phases (solubilization), phase II (conjunction) and phase III (excretion), each with its own enzymes (Krempl et al., 2016; Stahl et al., 2018; Lu et al., 2021). For example, cytochrome P450 monooxygenases (P450s) and carboxylesterases (CarE) carry out phase I, glutathione S-transferases (GSTs) and UDP-glycosyltransferases (UGTs) phase II, and ATP-binding cassette transporters (ABC) phase III (Nauen et al., 2022; Kennedy and Tierney, 2013; Jin et al., 2019; Ullah et al., 2020). These enzymes also work on endogenous substrates like hormones and lipids to carry out additional physiological processes and housekeeping functions in insects (Ketterman et al., 2011; Feyereisen, 2012). Host plant utilization and dietary diversity have both been linked to elevating detoxification enzyme activity and mRNA expression levels of related genes (Adesanya et al., 2016; Jin et al., 2019; Israni et al., 2020). In previous research, the increased P450 and GST activity have been observed in lepidopteran larvae and plant-feeding hemipterans feeding on non-preferred or less compatible plant species (Krieger et al., 1971; Yu, 1983; Mullin, 1986; Adesanya et al., 2016; Hafeez et al., 2021a). Similarly, higher P450 enzyme activity was observed in the generalist caterpillar *Spodoptera eridania* after feeding on the carrot, a non-preferred host plant compared to the lima bean, which is a more favored host (Brattsten, 2012). In addition, a significant variation in P450, CoE and GST activities in *Bemisia tabaci* and *Popillia japonica* have been reported among different host plants that are diverse in suitability (Xie et al., 2011; Adesanya et al., 2016). More recent research suggests that a large number of P450 genes related to detoxification enzymes from insects have been isolated and characterized (Schuler, 2011; Berenbaum and Calla, 2021; Berenbaum et al., 2021; Calla, 2021). For example, gossypol-induced P450s genes *CYP9A12*, *CYP9A14*, and *CYP9A98* showed high divergence in the mRNA level of *Helicoverpa armigera* and *Spodoptera exigua* larvae (Tao et al., 2012; Hafeez et al., 2019). *CYP6B8* and *CYP321A1* in the corn earworm, *Helicoverpa* zea, can metabolize xanthotoxin, flavone, quercetin as well as a variety of other phytochemicals, indicating that this insect species has evolved systems for phytochemical detoxification (Sasabe et al., 2004; Rupasinghe et al., 2007). Additionally, *CYP6B1* and *CYP6B3* in *Pailio polyxenes*, which specializes on Rutaceae and Apiaceae, show high efficiency in metabolism of furanocoumarins in its host plants and *CYP6AS* could metabolize the flavonoid quercetin in *Apis mellifera* (Mao et al., 2009). However, it is important to investigate how selective or inducible enzyme systems could provide generalist herbivores with an ecological opportunity to utilize secondary plant compounds before expending metabolic costs for detoxification.

The fall armyworm (FAW), *Spodoptera frugiperda* is a damaging insect that feeds on a variety of essential food crops, including maize and rice (Machado et al., 2008; Gouin et al., 2017; Hafeez et al., 2021a). Since 2016, this invasive pest has spread throughout Sub-Saharan Africa, resulting in significant agricultural losses (Goergen et al., 2016; Day et al., 2017; Kenis et al., 2022) and it has also made its way into South Asia, including China, where it is also dispersing rapidly across the region (Swamy et al., 2018; Li et al., 2020). Two ecological strains of FAW have been recognized from natural populations, the so-called corn and rice strains (Pashley, 1986; Nagoshi et al., 2019). Corn strain insects are prevalent on grasses such as maize and sorghum, while, insects belonging to the rice strain appear to predominate on small grasses such as rice and Bermuda grass. Although the two strains are identical physically in the field, they do have distinct preferences for host plants and show signs of reproductive isolation (Groot et al., 2010; Dumas et al., 2015). The recent genome sequencing of this species has provided new insights into how P450s function *in vivo* and how these enzymes and related genes may be involved in the pest insect’s adaptive mechanism (Gouin et al., 2017). Yet, the P450 enzymes and related genes induced by this polyphagous pest insect for host plants adaptation have not been characterized.

In this study, molecular characterization and functional analysis of cytochrome P450-mediated detoxification gene involved in host plant adaptation in *S. frugiperda* was examined after feeding on rice and corn host plants for consecutive 33 generations. Tissues and stage expression patterns of the CYP302A1 gene were also evaluated. Additionally, we investigated how cytochrome P450-specific detoxification enzyme led to larval mortality by PBO inhibitor followed by feeding on rice and corn host plants. To determine if the CYP302A1 gene functions in *S. frugiperda* host plant adaptation, a functional study of the gene was carried out using RNA interference.

## Materials and methods

During August 2019, larvae of *S. frugiprda* populations were collected from two different corn fields in Ping Hu, Zhejiang Province and maintained on corn seedlings in a climate control chamber at 25 ± 2°C with a 14: 10 h light: dark photoperiod at Zhejiang Academy of Agricultural Sciences, Hangzhou, China according to (Hafeez et al., 2021a).

### Reagents

7-Ethoxycoumarin, 7-hydroxycoumarin, NADPH, and Piperonyl butoxide were obtained from Sigma-Aldrich (St Louis, MO, USA). Bovine serum albumin was purchased from Thermo Scientific (Meridian Rd., Rockford, IL 61101, USA).

## Insect rearing and host plant selection

Two populations were established to avoid any homogenization effect. According to our previous study, the population was reared on corn plants for 33 generations (Hafeez et al., 2021a). Larvae for control treatment were reared on an artificial diet (Poitout and Bues, 1974). Both colonies were maintained in climate chambers at 27 ± 2°C and 70%–75% relative humidity (R.H) under a 14: 10 light: dark photoperiod until adult emergence. Each population was assigned a code denoting its host plant (corn or rice).

### Phylogenetic and bioinformatics analysis

We selected CYP302A1 as the representative gene to work on it based on our previous research work (Hafeez et al., 2021a). The protein sequence of *S. frugieprda* CYP302A1 was compared to other insects’ publicly released protein sequences using Protein BLAST: search protein databases using a protein query (nih.gov). Based on the amino acid sequence, the protein isoelectric point (pI) and molecular mass (kDa) were calculated using ExPASy: get pI/Mw. The MEGA 7.0 software (MEGA, Tempe, AZ, USA) was used to create a phylogenetic tree based on multiple alignments of protein sequences performed by ClustalW and using the neighbour-joining algorithm with bootstrap values determined by 1000 replicates.

### Tissues and stages expression analysis of CYP302A1 by RT-qPCR

The differential mRNA expression level of the CYP302A1 gene in different tissues of *S. frugiperda* larvae was analyzed after feeding on rice and corn host plants. Samples such as midguts, fat bodies were collected from larvae after feeding on rice, corn host plants and an artificial diet as a control treatment for 72 h. Similarly, thoraxes, heads and wings were collected from three days old adults respectively. A total of 30 individuals were selected from each treatment with three biological replicates (10 individuals per biological replicate). Three biological replicates were used for each experiment. Total RNA was extracted separately from all tissues (midguts, fat bodies, thoraxes, heads as well as wings from larvae and adults of *S. frugiperda)* using 1mL of TRIzolTM (Invitrogen, Carlsbad, CA, USA) and cDNA was prepared from total RNA using TransScript^®^ OneStep gDNA Removal and cDNA Synthesis SuperMix according to the manufacturer’s instructions. The primer sequences used for the candidate gene are listed in **Table S1**. Three biological replicates and three technical replications for each cDNA sample were used for RT-qPCR analysis. The CFX96TM Real-Time PCR Detection System (Bio-Rad Hercules, CA, USA) with the iTaq Universal SYBR Green Supermix (BIO-RAD according to the manufacturer’s instructions was used for RT-qPCR analysis. The relative levels of mRNAs were quantified using three biological replicates and normalized using GAPDH (GenBank: KC262638.1) and S30 (AF400225.1) as an internal control according to the protocol described by (Bustin et al., 2009). The fold changes were determined using the 2^−ΔΔCt^ method followed (Livak and Schmittgen, 2001).

### Measurement of P450 enzyme activity

#### Midguts collection and sample preparation

The midguts of the larvae were dissected at 48, 72 and 96 h after feeding on the corn, rice plant and an artificial diet (Ck) as a control treatment according to (Hafeez et al., 2021b). Larvae from each treatment were cold immobilized and their midguts were separated in 0.1 mol/L phosphate-buffered saline with pH 7.4. A total of 30 larval midguts were dissected and pooled for metabolic activity experiments for each biological replicate and stored at −80°C until enzymatic activity assays were performed.

#### Enzymatic activity of P450

Evaluation of P450 enzyme activity was carried out following the protocol described by (Chen et al., 2018) with some minor adjustments. According to the method described by (Chen et al., 2018), the midguts enzyme activity of *S. frugiperda* larvae was measured using 7-ethoxycoumarin (7-EC) as the substrate. The midguts of thirty *S. frugiperda* 4th-instar larvae were homogenized on ice with two millilitres of homogenization buffer 0.1 M PBS at pH 7.5. The supernatant from tubes of 2 millilitres that had been subjected to centrifugation was collected and then used for P450s activity assay. Immediately after the reaction, the concentration of 7-hydroxycoumarin in the reaction mixture was determined by employing a SPECTRA max GEMINI XS spectrofluorometer (Molecular Devices, USA) and adjusting the excitation and emission filters to 356 nm and 465 nm, respectively. At least three separate experiments were carried out for each biochemical analysis with different preparations of enzymes. The method described by (Bradford, 1976) was utilized to get the results for the protein concentration using bovine serum albumin as the standard protein. The activity was recorded as nmol p-nitroanisole/min/mg protein.

#### Effect of piperonyl butoxide (PBO) on larval mortality after feeding on host plants

To further confirm the possible role of metabolic detoxification enzyme in *S. frugiperda* larvae to host plant adaptation. PBO solution at the concentration of 50 mg/L was prepared in 1% (v/v) acetone. For both rice and corn groups, acetone solution containing (1 μL) of PBO was applied to the pronotum of each third instar larvae and 1% (v/v) acetone only as a control using a hand applicator. Following a post-treatment with PBO for twenty-four hours, the larvae were transferred to the host plant treatments. Each independent plant treatment (15-d-old corn and rice seedlings) had a total of 50 *S. frugiperda* larvae with 10 replicates (5 larvae per replicate). After feeding on different rice and corn plants and control, the mortality and larval mass were recorded at 48, 72, and 96 h. Each experiment was triplicated.

### Processes of preparing, quantifying, and purifying double-stranded RNA

For dsRNA synthesis, CYP302A1 with a fragment size of 365bp was amplified by PCR. The primers used for the CYP302A1 amplifications were designed to add the T7 polymerase promoter sequence to the 5 ends of each strand (**Table S1**). Similarly, the DEPC-treated water was used as a control treatment. The dsCYP302A1 template, which was generated through PCR and then purified, was prepared following the instructions included in the T7 RiboMax Express RNAi System Kit (Promega, Madison, WI, USA). The MEGAclearTM Kit (Ambion, Austin, TX, USA) was used to purify the resulting dsRNA. The quality of dsRNA was confirmed using 1.5% agarose gel electrophoresis, and the concentration of the final dsRNA of the target gene was measured using a NanoDrop^®^ spectrophotometer (Thermo Fisher, Waltham, MA, USA), then the final dsRNA solution was frozen at -80 degrees Celsius until to use.

### dsRNA feeding bioassays for mortality and larval growth

In this study, we used the droplet-feeding method for RNAi to prevent damage to *S. frugiperda* larvae as previously defined by (Wang et al., 2018b; Hafeez et al., 2022). The dsRNA was first diluted (250 μg/μL total volume of 500μL) in diethylpyrocarbonate (DEPC)-treated water before dsRNA feeding experiments. One-day-old 3rd-instar larvae from the Corn and Rice populations were starved for 6 h before use for feeding bioassays. Starved larvae were moved individually in sterilized 24-well tissue culture plates with 1 g of artificial diet. A total of 1μL dsRNA solution (250 μg/μL) of target CYP302A1gene was placed at the centre of each well using a 2- μL pipette. After 24 h on an artificial diet with dsRNA solution, larvae were transferred to host plant treatments. Similarly, the plants treated with DEPC-treated water were used as a control treatment. Each independent plant treatment had a total of 50 *S. frugiperda* larvae with 10 replicates (5 larvae per replicate). The larvae were transferred onto 15-d-old corn and rice seedlings. For mortality analysis, the mortality was recorded at 48, 72 and 96 h and total larval duration was assessed until pupation after feeding on different rice and corn treatments and control. Each experiment was repeated in triplicate.

### RNA extraction and cDNA preparation for RT-qPCR

The differential expression and knockdown of the CYP302A1 gene were studied to validate the function of CYP302A1 in host plants adaptation. Larvae were fed on an artificial diet containing dsRNA solution for 24 h, then transferred to the host plant and control treatments for 48, 72 and 96 h. A total of 24 individual larvae were selected and 8 larvae served as a biological replicate for each treatment. Three independent biological replicates were used for each experiment as described above.

### Statistical analysis

SPSS 13.0 Software Package (SPSS Inc., Chicago, IL, USA) was used to analyze all data including larval weight, larval growth, enzyme activity and transcript levels of the CYP302A1 gene. Statistically significant differences were determined by Student t-test and one-way analysis of variance followed by Tukey’s HSD multiple comparison tests (*P* < 0.05).

## Results

### Expression Profiling of the P450 Gene at Developmental Stages and Tissues

**Figure 1:**
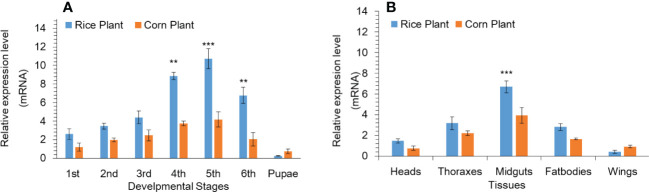
Developmental **(A)** and tissue-specific **(B)** expression pattern of *Spodoptera frugiperda CYP302A1* after feeding on rice and corn plants. Real-time quantitative RT-qPCR analysis was used to determine relative transcript levels. Data shown are mean ± SE. Treatments were compared using Student’s t test. ** and *** represent P < 0.01 and P < 0.00, respectively.

Based on our previous research work (Hafeez et al., 2021a), we found that P450 genes shows high expression. Among all the upregulated P450 genes, we found that the CYP302A1 gene was a highly up-regulated as compared to other P450 genes as well as FPKM values showed the same expression pattern as compared to other genes among different treatments (**Table S2**). The mRNA expression level of the P450 gene CYP302A1 at various developmental stages and different tissues of *S. frugieprda* was measured after rearing on rice plants and corn plants for 33 generations respectively (**Figure 1A**). Results indicated that the expression level of the P450 gene CYP302A1 was the highest in fifth-instar (rice plants; 10.7-fold) larvae among developmental stages followed by fourth-instar larvae (rice plants; 8.8 and corn plants; 3.7-fold), compared with the corn plants (5^th^ instar; 4.2-fold and 4^th^ instar; 3.7-fold. whereas the expression level of the CYP302A1 gene was the lowest at the first-instar (2.6-fold) and pupal stage (0.25-fold) after rearing on rice plants for 33 generations in comparison with corn plants (**Figure 1A**).

In addition, we analyzed the tissue distribution expression level of P450 CYP302A1 gene (**Figure 1B** **)**. The midguts and fat bodies were dissected from fourth instar larvae and the different tissues were taken from 3-d old adults (heads, thoraxes and wings) after rearing on rice and corn plants for 33 generations respectively. The results showed that the mRNA expressed level of CYP302A1 was the highest in the midguts followed by fat bodies (6.7 and 2.8-fold) after rearing on rice plants compared with midguts and fat bodies (3.9 and 1.6-fold) after rearing on rice plants (**Figure 1B**). Whereas, the expression level of CYP302A1 was the highest in thoraxes and heads with 3.1- and 2.2-fold in the rice population compared with thoraxes and heads of the corn population respectively (**Figure 1B**).

### P450 enzyme activity assays of 4^th^ instar larvae

**Figure 2:**
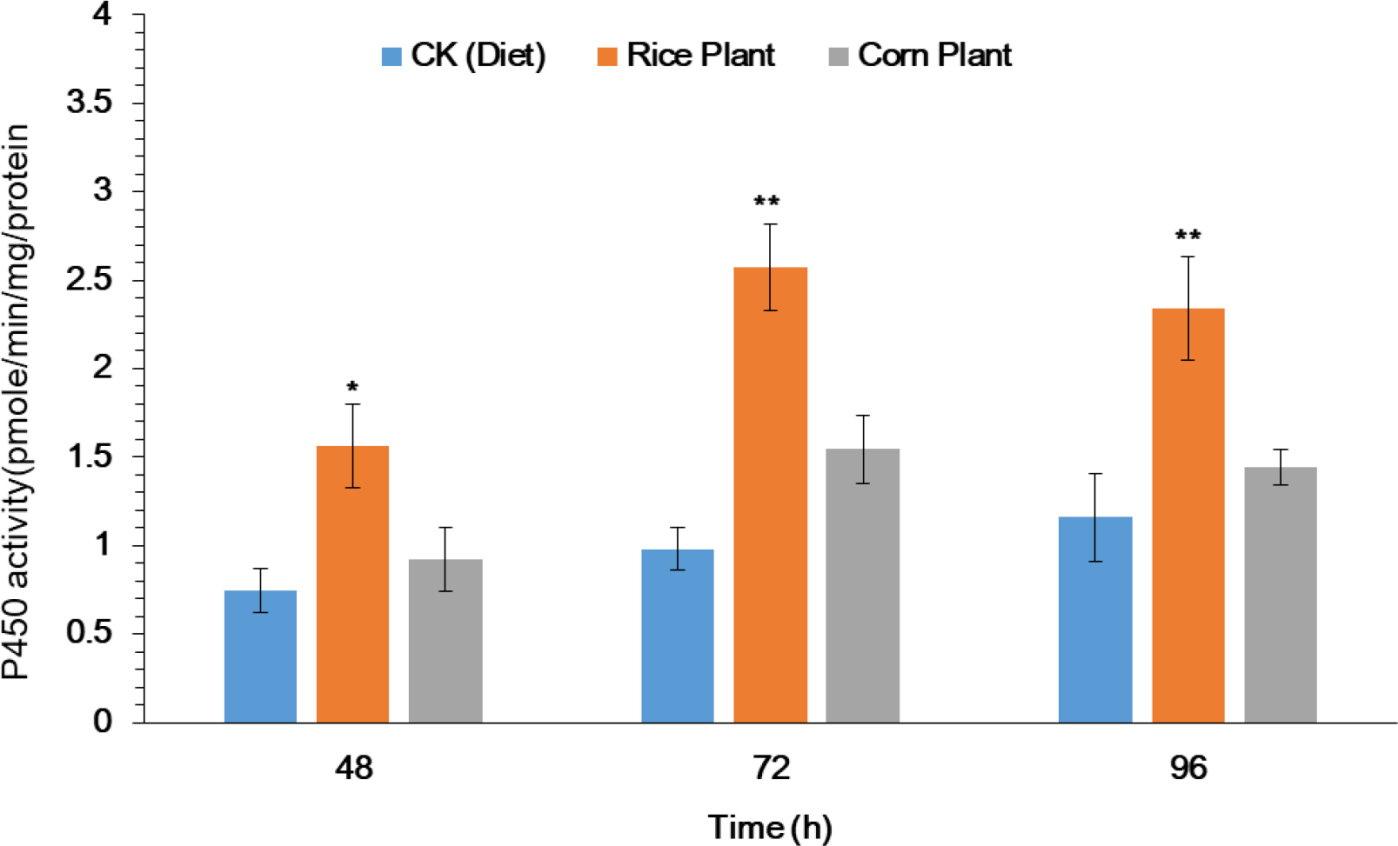
Activity of P450 enzyme in midguts of the fourth-instar larvae of FAW after feeding on rice and corn plants. The data were expressed as the means ± SE. Treatments were compared using Duncan’s multiple range test. * and ** represent P < 0.05 and P < 0.01, respectively.

The purpose of this study was to investigate the potential role that metabolic detoxification enzyme plays in the host plant adaptation mechanism of *S. frugiperda* larvae. After 33 generations of rearing on rice and corn plants, the activity of the cytochrome P450 enzyme (P450s) in the midguts of 4th instar *S. frugiperda* larvae was evaluated. Significantly enhanced P450 activity was observed in midguts of 4^th^ instar larvae after exposure to rice seedlings as compared to corn seedlings and artificial diet (**Figure 2**). After exposure to rice and corn plants, significantly increased activity of P450 enzyme was observed in the midguts by 1.56, 2.56 and 2.38 as compared to corn plants 0.92, 1.21 and 1.34 at 48, 72 and 96h respectively (**Figure 2**). However, the results suggest that the P450 enzyme plays a significant role in the adaptation of *S. frugiperda* to its host plant.

### The effect of piperonyl butoxide (PBO) on larval mortality

**Figure 3:**
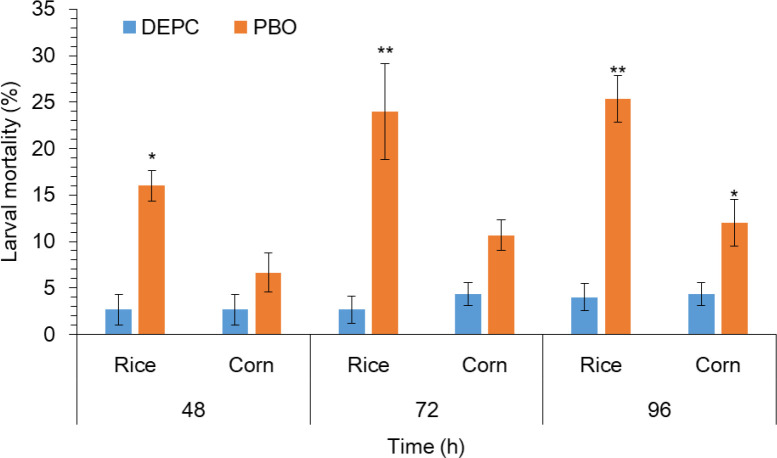
The impact of piperonyl butoxide (PBO) on larval mortality of FAW. 1 μL of PBO was applied to the pronotum of each third instar larvae and 1% (v/v) acetone only as a control using a hand applicator for 24 h followed by feeding on rice and host plants. After feeding on different rice and corn treatments and control, mortality and larval mass were recorded at 48, 72, and 96 h. Data shown are mean ± SE. Treatments were compared using Student’s t test. * and ** represent P < 0.05 and P < 0.01, respectively.

To further confirm the possible role of metabolic detoxification enzyme in *S. frugiperda* larvae to host plant adaptation. PBO, a known inhibitor of the P450 enzyme was added to the diet and fed to third-instar larvae for 24 h followed by the exposure of rice and corn plants. Results indicated that the mortality of larvae pretreated with PBO was even significantly higher on rice plants as compared to the corn plant (**Figure 3**). Whereas, the trend of mortality was significantly higher in larvae pretreated with PBO followed by the exposure of rice plants at 72 and 96 h as compared with the larvae without treated of PBO followed by the exposure of rice plants respectively (**Figure 3**). This result suggesting a vital role of P450s in rice plant adaptation in *S. frugiperda.*

### *CYP302A1* gene characterization and phylogeny

**Figure 4:**
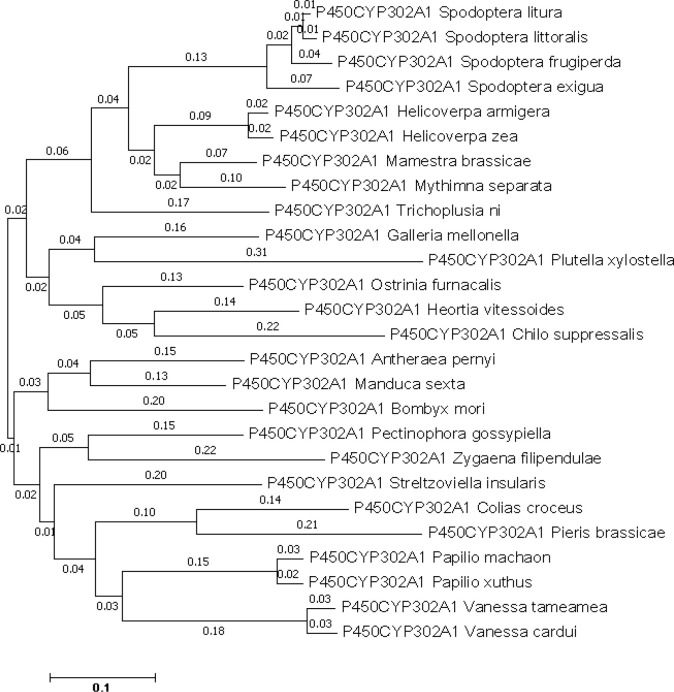
Phylogenetic analysis of *Spodoptera frugiperda* CYP302A1 and related P450s. A phylogenetic tree was constructed by the neighbor joining (NJ) method using Mega 7.0 software. The scale bar indicates 0.1 amino acid substitutions per site. Bootstrap analysis was performed with 1000 iterations.

The *CYP302A1* sequence with an open reading frame (ORF) of 1518 bp long, which encodes a protein of 495 amino acid residues. According to the translated amino acid sequence, *CYP302A1* has a theoretical pI value of 8.873 and a predicted mass of 58.84 kDa. The alignment of the deduced amino acid sequence of *S. frugiperda CYP302A1* with members of the *CYP321* family from other insect species demonstrated that it possesses the conserved motifs and conserved domains that are present in other P450 members (**Supplementary Figure 1**). In the comparison between *S. frugiperda CYP302A1* and the putative amino acid sequences of *Mamestra brassicae*, *Spodoptera litura*, *Spodoptera littoralis*, and *Spodoptera exigua*, the *S. frugiperda* sequence shared the highest level of similarity. (**Figure 4**).

### Silencing of CYP302A1 by dsRNA

**Figure 5:**
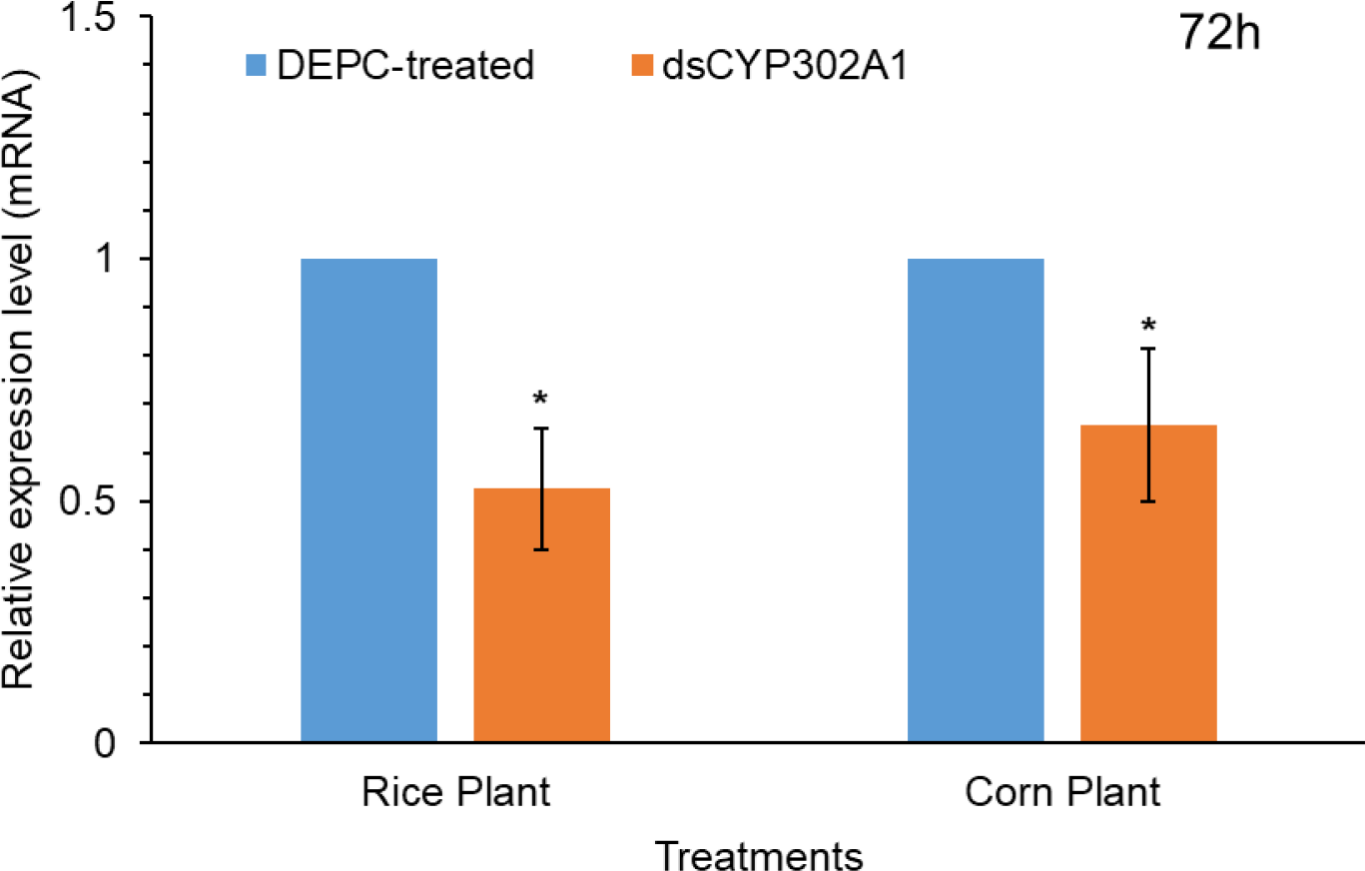
The relative mRNA transcript levels in the midguts of *S. frugiperda* larvae after feeding on dsCYP302A1 DEPC-treated water for 72 h. Data shown are mean ± SE. Treatments were compared using Student’s t test. * represent P < 0.05.

To assess whether the knockdown of detoxification *CYP302A1* gene of *S. frugiperda* plays a significant role in host plant adaptation (**Figure 5**). RNA-mediated down-regulation of this gene was evaluated using early third-instar larvae feeding on rice and corn plants. Pretreated larvae with dsRNA and DEPC-water treated plants as control *via* droplet feeding using an artificial diet for 24 h followed by feeding on rice and corn plants. Results showed significant down-regulation of the expression levels of the *dsCYP302A1* gene in the *S. frugiperda* larvae after feeding on rice and corn plants compared with the DEPC-water treated plants as control at 72 h (**Figure 5**).

### Silencing effect of dsCYP302A1 on larval mortality, larval duration and weight gain

**Figure 6:**
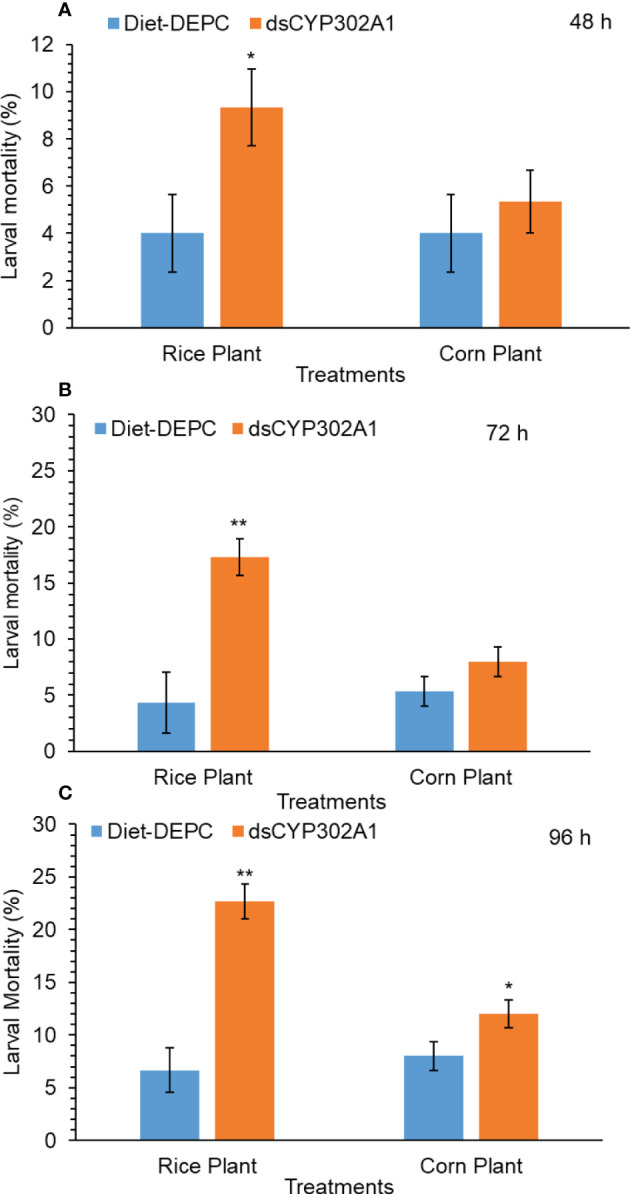
Larval mortality of S. frugiperda larvae after treated with diet containing dsCYP302A1and DEPC-treated water followed by feeding on rice and host plants for 48 **(A)**, 72 **(B)** and 96 h **(C)**. Data shown are mean ± SE. Treatments were compared using Student’s t test. * and ** represent P < 0.05 and P < 0.01, respectively.

Results indicated that the down-regulation of the *dsCYP302A1* significantly increased mortality of *S. frugiperda* larvae when larvae were pretreated with dsRNA for 24 h followed by feeding on the rice plants compared with the DEPC treated water plants as a control treatment for 48 h (**Figure 6A**). On the other hand, no larval mortality was observed of the corn plants when larvae were pretreated with dsRNA for 24 h followed by feeding on the corn plants compared with the DEPC treated water plants as a control treatment for 48 h (**Figure 6B**). Similar trend was observed in larval mortality when larvae were pretreated with dsRNA for 24 h followed by feeding on the rice plants compared with the DEPC treated water plants as a control treatment for 72 h and 96 h respectively (**Figure 6C**). Whereas, no significant larval mortality was found when larvae were pretreated with dsRNA for 24 h followed by feeding on the corn plants as compared with the DEPC treated water plants as a control treatment for 72 h and 96 h (**Figure 6C**).

**Figure 7:**
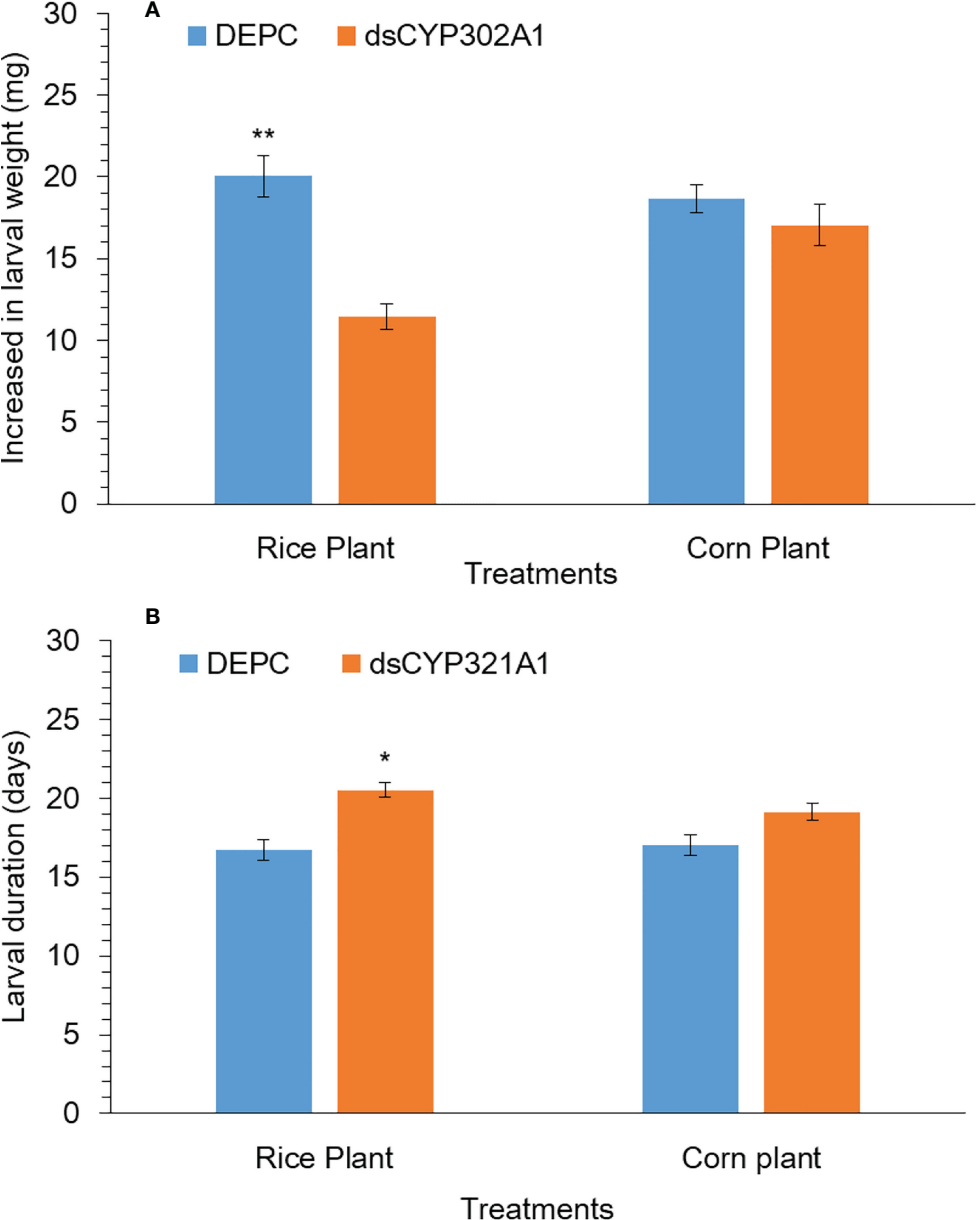
Larval weight **(A)** and larval duration **(B)** of S. frugiperda larvae after treated with diet containing dsCYP302A1and DEPC-treated water followed by feeding on rice and host plants. Data shown are mean ± SE. Treatments were compared using Student’s t test. * and ** represent P < 0.05 and P < 0.01, respectively.

The silencing effect of the *CYP302A1* gene on the *S. frugiperda* larval development and weight gain was evaluated after exposure to a dsRNA-treated diet and DEPC-treated diet as a control for 24 h followed by the feeding rice and corn plants. Our results showed that larval duration significantly increased when early third instar larvae were exposed to dsRNA-treated diets of dsCYP302A1 for 24 h followed by the feeding rice plants at 72 h (**Figure 7A**). While, no significant effect on larval duration was observed when early third instar larvae exposed with dsRNA-treated diets of dsCYP302A1 for 24 h followed by the feeding corn plants at 72 h (**Figure 7A**). Similarly, significantly decreased of the larvae weight gain was found when early third instar larvae exposed with dsRNA-treated diets of dsCYP302A1 for 24 h followed by the feeding rice plants at 72 h (**Figure 7B**). While no significant effect on larval weight gain was observed when early third instar larvae exposed with dsRNA-treated diets of dsCYP302A1 for 24 h followed by the feeding rice plants at 72 h (**Figure 7B**). Further results indicated that fewer rice plants were consumed when early third instar larvae were exposed to dsRNA-treated diets of dsCYP302A1 for 24 h followed by the feeding rice plants compared with corn plants and DEPC treated diet as control at 72 h (**Supplementary Figures 2A–D**).

## Discussion

Herbivorous insects and their host plants use signals from each other to intensify reciprocal responses (Rashid War et al., 2018; Yactayo-Chang et al., 2020a). Upon attack by herbivorous insects, plants increase the synthesis of defensive compounds such as phytochemicals and proteins to fend them off (Zhu-Salzman and Zeng, 2015; Yactayo-Chang et al., 2020b). In response, herbivores have to evolve diverse strategies to overcome several challenges, to thrive on chemically defense compounds present in their host plant tissues by increasing the activity of their counteroffensive digestive and detoxification mechanism (Heidel-Fischer and Vogel, 2015b; Rashid War et al., 2018; Hafeez et al., 2021b).

In this study, we reported the characterization and functional analysis of the detoxification gene *CYP302A1* and how *S. frugieprda* larvae use a detoxification mechanism to adapt host plants. The functional and evolutionary diversification of insect P450s was likely a key factor in accelerating the unprecedented success of insects (Feyereisen, 2006b; Zhu et al., 2018). The deduced amino acid sequence of *S. frugiperda CYP302A1* aligned with other insect *CYP321* family members showed that it has conserved motifs and domains (Wang et al., 2017). In previous studies, it has been shown that P450 expression profiles vary dramatically during different developmental stages in most insects (Feyereisen, 2006b; Wang et al., 2018a). Similarly, it has been reported that the midgut fat bodies and Malpighian tubules are frequently associated with higher P450 activity (Hu et al., 2014;). In addition, the midgut is a highly crucial organ in the process of detoxification, and the genes that code for the detoxification enzymes involved in this process are frequently highly regulated in this organ (Feyereisen, 2006a; Hafeez et al., 2022). In this study, we investigated the expression profile of *CYP302A1* in different tissues and developmental stages of *S. frugiperda* by quantitative RT-qPCR. Similar to the expression patterns of *CYP6B48*, *CYP658*, and *CYP321B1* in *S. litura* larvae, which may potentially be involved in the plant allelochemicals metabolism, our findings demonstrated that *CYP302A1* expression levels were much higher in the midgut tissue and the older *S. frugiperd*a larvae. Similarly, the elevated expression level of *CYP321E*, *CYP321A8*, *CYP321A9*, and *CYP321B1* genes was reported in midguts and fat bodies of *P. xylostella* and *S. frugiperda* (Bai-Zhong et al., 2020). The enhanced expression level observed in midgut tissue and late larval instars could be attributed to a greater need for xenobiotic detoxification at this stage due to increased feeding activity (Feyereisen, 2006a; Wang et al., 2015; Israni et al., 2020). Tissue-specific expression levels of the *CYP302A1* gene in *S. frugiperda* further suggest that *CYP302A1* could be involved to adapt the host plant by detoxifying the plant xenobiotics.

Utilization of a large diversity of host plants and diet variability increase detoxifying enzyme activity in insect herbivores (Ahmad, 1983; Adesanya et al., 2016; Erb and Reymond, 2019). Elevated enzyme activities may be induced by the variety of phytochemicals present across the host pant range (Feyereisen, 2005). Our current studies revealed the enhanced P450 activity in the midguts of *S. frugiperda* larvae after exposure to rice plants as compared to corn plants and an artificial diet. Our findings are consistent with those of earlier studies; for instance, consumption of a non-preferred host plant by caterpillars of the *Spodoptera eridania* species and adult Japanese beetles induced higher P450 enzyme activities in comparison to the consumption of a more preferred host plant (Brattsten, 2012; Adesanya et al., 2016). Similarly, variation in P450 activities among five host plants that varied in suitability has been reported in *Bemisia tabaci* (B-biotype), a generalist whitefly (Xie et al., 2011). To further confirm the possible role of metabolic detoxification enzyme in *S. frugiperda* larvae to host plant adaptation, higher mortality was observed in pretreated larvae with PBO followed by the exposure of rice plants as compared to the corn plants. Our results are consistent with (Wu et al., 2021) who reported higher mortality of *H. armigera* larvae pretreated with PBO after exposure to plant volatile than control. Though, selective or inducible enzyme systems could provide generalist herbivores with an ecological opportunity to adapt to diverse host plants. This could be accomplished by generalist herbivores using secondary compounds present in their host plants before expending metabolic resources on detoxification.

Functional analysis of important genes has extensively been studied in insects using the RNAi technique (Kim et al., 2015; Choi and Vander Meer, 2019; Adeyinka et al., 2020; Hafeez et al., 2021b; Ullah et al., 2022). To further investigate if the host-plant-induced gene CYP302A1 is involved in host-plant adaptation, we fed dsCYP302A1 to *S. fugiperda* larvae to study the knockdown effects of the target gene on mortality and growth parameters. The dsRNA of target gene-fed larvae significantly showed reduced *CYP302A1* mRNA expression level in the midgut followed by the exposure of rice plant as compared to the corn plant and DEPC-water treated plants as a control. Similar to our findings, RNAi-triggered *CYPAB14*, *CYPA98*, *CYP321A7* and *CYP6B8* genes downregulation through uptake of dsRNAs have been documented for other insect species (Li et al., 2000; Mao et al., 2011; Hafeez et al., 2019; Hafeez et al., 2022). Present results indicate that the dsRNA-mediated knockdown of *CYP302A1* in the *S. frugieprda* larvae lead to higher mortality after feeding rice plant as compared to the corn plant and DEPC-water treated plants as control at 72 and 96 h. Our results provide the advocacy of previous findings by (Hafeez et al., 2019) who reported that silencing of *CYP6AB14* and *CYP9A98* genes in *S. exigua* larvae followed by feeding on 0.1% gossypol caused larval mortality. Similarly, silencing of HaAK gene in *H. armigera* using RNAi-mediated transgenic plant increased larval mortality when larvae were fed on the leaves of the transgenic plant (Liu et al., 2015). In previous reports, it has been documented that silencing of the *CYP6AE14* gene in *H. armigera* larvae and *CYP6AB14* and *CYP9A98* genes in *S. frugiperda* larvae by transgenic plant-mediated RNAi retarded larval growth and weight (2011; Mao et al., 2007; Tao et al., 2012; Hafeez et al., 2022). Similarly, the results obtained in the present work also indicated that the RNAi-mediated knockdown of the CYP302A1 gene increased larval mortality, reduced the larval weight and developmental time after exposure to a dsRNAs-supplemented diet with subsequent feeding on host plants as compared to the control.

## Conclusion

In this study, we provide evidence that the insect P450 monooxygenases play a key role in host plant adaptation by detoxifying plant defense compounds. In the current study, tissue-specific expression levels of the CYP302A1 gene in *S. frugiperda* further advocate that CYP302A1 might be involved to adapt host plants. We revealed the enhanced P450 activity in the midguts of *S. frugiperda* larvae after exposure to rice plants as compared to corn plants and an artificial diet. These results concluded that the inducible enzyme system and related genes, however, could provide herbivores with an ecological opportunity to adapt diverse host plants by utilizing phytotoxins present in their host plants. We reported that the dsCYP302A1 caused mortality and had harmful effects on the growth and development of *S. frugiperda* larvae before exposure to dsRNA followed by the feeding on host pants. The harmful effects would be magnified if RNAi targeted multiple genes involved in the P450 complex system. Further studies are needed to explore more P450 genes using RNAi-based approaches against insect pests for crop protection based on a recently developed genetic tool.

## Data availability statement

The original contributions presented in the study are included in the article/**Supplementary Material**. Further inquiries can be directed to the corresponding authors.

## Author contributions

The original study design was made by MH, XL, YGL and YL and discussed with the other authors and approved the manuscript All authors contributed to the article and approved the submitted version.
