# Supplementary material for: Molecular characterization and functional analysis of cytochrome P450-mediated detoxification CYP302A1 gene involved in host plant adaptation in Spodoptera frugieprda
Source: Front Plant Sci. 2023 Jan 25;13:1079442. doi: 10.3389/fpls.2022.1079442 (PMC9906809; doi:10.3389/fpls.2022.1079442)
Supplement: Supplementary file 1 [file DataSheet_1.pdf]

Molecularcharacterization and functional analysis of cytochrome P450-mediated detoxification CYP302A1 gene involved in host plant adaptation in *Spodoptera frugieprda*

Muhammad Hafeez[1,2] Xiaowei Li[2], Limin Chen[2,6] Farman Ullah[3] Jun Huang[1] Zhijun Zhang[2] Jinming Zhang[2] Junaid Ali Siddiqui[4] Shu-xing Zhou[2] Xiao-yun Ren[2] Muhammad Imran[5] Mohammed A. Assiri[5] Yonggen Lou[1]* and Yaobin Lu[2]*

[1] State Key Laboratory of Rice Biology and Ministry of Agriculture Key Lab of Molecular Biology of Crop Pathogens and Insects, Institute of Insect Sciences, Zhejiang University, Hangzhou, China
[2] State Key Laboratory for Managing Biotic and Chemical Threats to the Quality and Safety of Agro-products Institute of Plant Protection and Microbiology, Zhejiang Academy of Agricultural Sciences, Hangzhou, China
[3] Department of Plant Biosecurity, College of Plant Protection, China Agricultural University, Beijing, China
[4] College of Agriculture, College of Tobacco Science, Guizhou University, Guiyang 550025, China
[5] Department of Chemistry, Faculty of Science, King Khalid University, P.O. Box 9004, Abha 61413, Saudi Arabia
[6] Integrated Plant Protection Center, Lishui Academy of Agricultural and Forestry Sciences, Lishui, China

*Correspondence:
1. Professor Yonggen Lou yglou@zju.edu.cn
2. Professor Yaobin Lu luybcn@163.com

Table S1. Primers used in this study for RTq-PCR and RNAi

Gene name	Sense primers	Anti-sense primers	
**dsCYP302A1**	5'- ggatcctaatacgactcactatag GAGACTGAGAAGCACATTTC -3'	5'-ggatcctaatacgactcactatag GCCATCTTCAGGTTGTTC-3	427
**RTq-PCR**	5'- TGAACAACCTGAAGATGGCG-3'	5'- AACCATGACTCCAATGACGTC-3	91
**S30**	CACCCTCGGTGTTAGACGTT	CCACCGGGAAAGTGATACTGT	119
**GAPDH**	CGGTGTCTTCACAACCACAG	TTGACACCAACGACGAACAT	111

The small capital letter shows the T7 RNA polymerase promoter sequence.

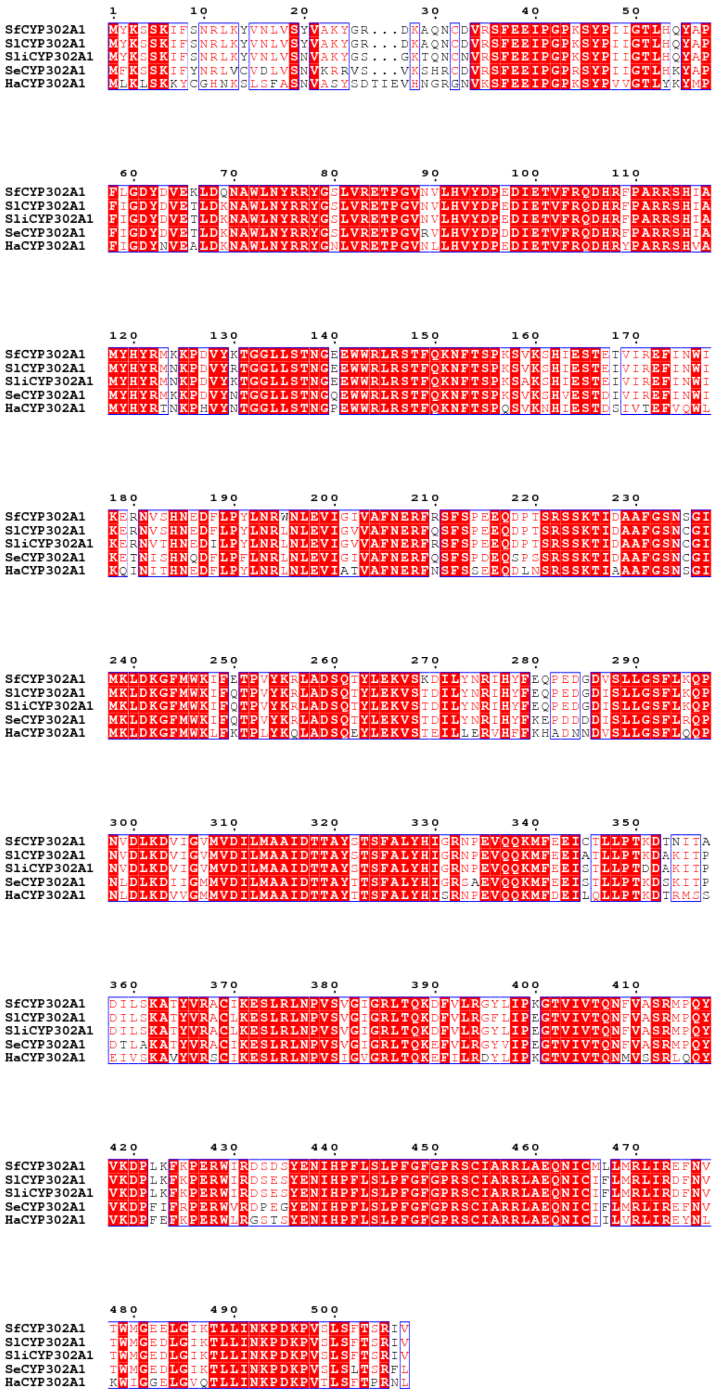

Fig. S1 Alignment of the deduced amino acid sequences of CYP302A1 (Spodoptera frugiperda), (Spodoptera litura), (Spodoptera littoralis), (Spodoptera exigua) and (Helicoverpa armigera). Identical amino acid residues, conserved residues and Conserved motifs of cytochrome P450 proteins are boxed.

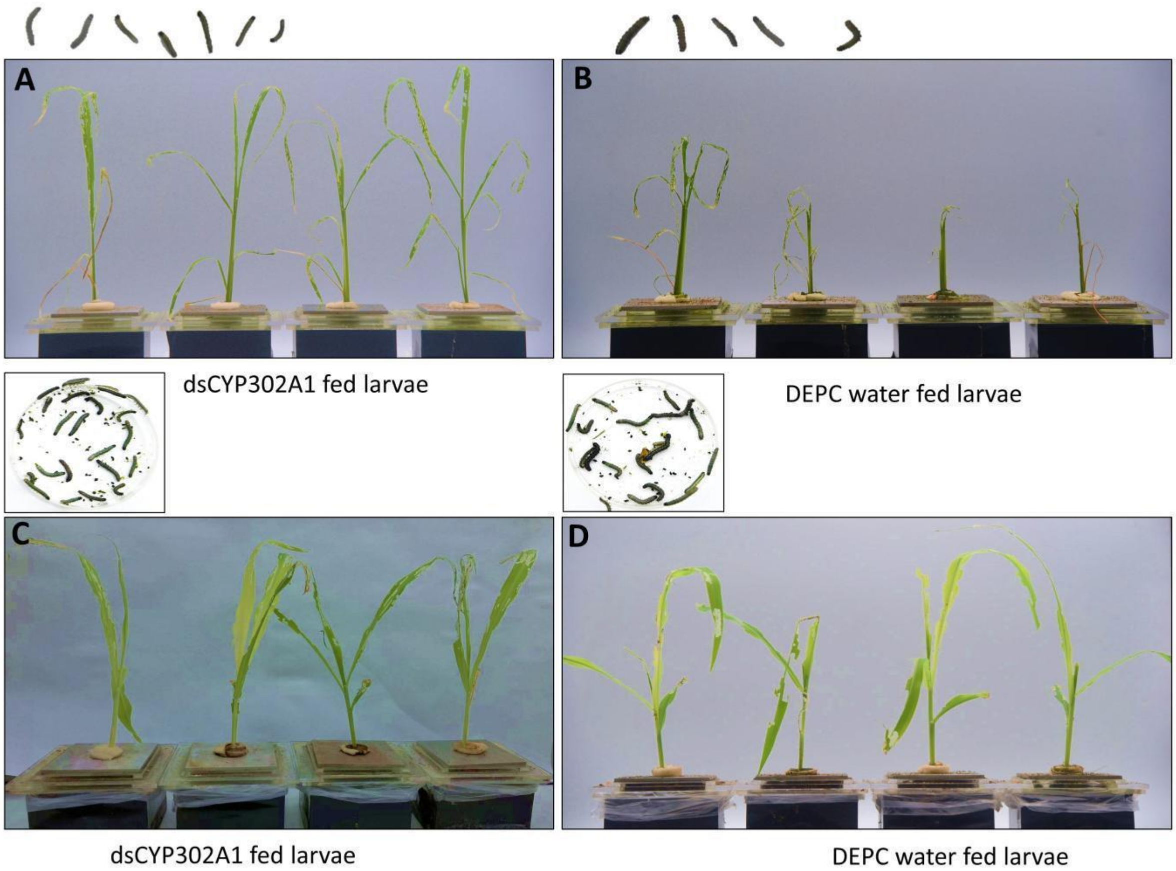

Fig. S2 A, B, C & D Consumption of rice plants (A) and corn plants (B) by *S. frugiperda* larvae after treated with diet containing dsCYP302A1and DEPC-treated water followed by feeding on rice and host plants.
