# Supplementary material for: Molecular characterization and functional analysis of cytochrome P450-mediated detoxification CYP302A1 gene involved in host plant adaptation in Spodoptera frugieprda
Source: Front Plant Sci. 2023 Jan 25;13:1079442. doi: 10.3389/fpls.2022.1079442 (PMC9906809; doi:10.3389/fpls.2022.1079442)
Supplement: Supplementary file 2 [file DataSheet_2.pdf]

Molecular characterization [i]and functional analysis of cytochrome P450-mediated detoxification CYP302A1 gene involved in host plant adaptation in *Spodoptera frugieprda*

Muhammad Hafeez1, 2, Xiaowei Li2, Limin Chen2,6, Farman Ullah3, Jun Huang1, Zhijun Zhang2, Jinming Zhang2, Junaid Ali Siddiqui4, Shu-xing Zhou2, Xiao-yun Ren2, Muhammad Imran5· Mohammed A. Assiri5, Yonggen Lou1* and Yaobin Lu2*

Table S2.

Rice-Mid vs Corn- Mid

Gene ID	log2FoldChange	gene description
GSSPFG00001269001	2.377173	PF00067: Cytochrome P450
GSSPFG00016942001.2	6.11486	PF00067: Cytochrome P450 CYP302A1
novel.1430	2.794915	PF00067: Cytochrome P450
GSSPFG00033556001.2	1.647941	PF00067: Cytochrome P450
GSSPFG00005009001.2	1.941901	PF00067: Cytochrome P450
GSSPFG00026573001.4	2.720602	PF00067: Cytochrome P450
GSSPFG00019627001	3.21476	PF00067: Cytochrome P450 CYP302A1
GSSPFG00033942001	3.878751	PF00067: Cytochrome P450
